# Selective compounds enhance osteoblastic activity by targeting HECT domain of ubiquitin ligase Smurf1

**DOI:** 10.18632/oncotarget.10648

**Published:** 2016-07-18

**Authors:** Yuan Zhang, Cheng Wang, Yu Cao, Yongqing Gu, Lingqiang Zhang

**Affiliations:** ^1^ State Key Laboratory of Proteomics, Beijing Proteome Research Center, Beijing Institute of Radiation Medicine, Collaborative Innovation Center for Cancer Medicine, Beijing 100850, China; ^2^ Department of Basic Medical Sciences, School of Medicine, Shihezi University, Shihezi, Xinjiang Province 832000, China; ^3^ Department of Neuroscience and Regenerative Medicine, Medical College of Georgia, Augusta University, Augusta, GA 30912, USA

**Keywords:** Smurf1, HECT domain, Ub binding region, ubiquitination, osteoblastic activity

## Abstract

The HECT-type ubiquitin ligase Smurf1 (Smad ubiquitination regulatory factor-1) plays the prominent role in regulation of bone formation, embryonic development, and tumorigenesis by directing the ubiquitin-proteasomal degradation of specific targets. In contrast with RING-type E3s, the catalytic HECT domain of Smurf1 firstly binds to and then transfers ubiquitin (Ub) molecules onto the substrates. The Smurf1-Ub interaction is required for Smurf1 catalytic ligase activity to promote substrate degradation. However, so far specific regulators or compounds controlling Smurf1-Ub interaction and the ligase activity have not been identified. Here we report two small molecule compounds targeting Ub binding region of HECT domain interrupt Smurf1-Ub contact, inhibit Smurf1 ligase activity and stabilize BMP signal components Smad1/5 protein level. Furthermore, these compounds increase BMP signal responsiveness and enhance osteoblastic activity in cultured cells. These findings provide a novel strategy through targeting Smurf1 ligase activity to potentially treat bone disorders such as osteoporosis.

## INTRODUCTION

Ubiquitination, which involves in the intracellular protein turnover of various biological processes, is essential for maintaining physiological function of organism and cellular homeostasis. The dysfunction of this process will lead to the disorders and disease of body, including cardiac dysfunction [1], bone degenerative disease [2], metabolic disorders [3], and cancer malignancy [4, 5]. During ubiquitination, E3 ubiquitin ligases function as the final link to recognize and select substrate for protein degradation [6]. More than 600 different ubiquitin-protein ligases have been identified [6], which are classified into two types: the scaffold-type and the thioester bond intermediate-type [7–9]. The vast majority of E3 ligases belong to the former type, which contains a RING (Really Interesting New Gene) finger domain, whereas the latter form the E6AP carboxyl terminus (HECT) domain family [8]. RING E3 ligases ubiquitinate and degrade targets in an E2-dependent manner, during which process, the E2s and target proteins interact with E3s respectively. By the medium of E3s, E2s transfer ubiquitin (Ub) directly to specific internal Lys residues of those proteins. That means Ubs are delivered from E2 to target proteins and tagged onto the specific lysine residue of substrates. Unlike RING E3s, the HECT E3s interact with E2s and are responsible for the delivery of Ub. HECT E3s receive Ub molecules by the conserved HECT domain in the C terminus and form the Ub-thioester to transfer Ub onto subtracts for ubiquitination [10].

Smurf1, a C2-WW-HECT ubiquitin ligase, which belongs to the Nedd4 (neural precursor cell expressed developmentally downregulated gene 4) family is a key negative regulator of transforming growth factor (TGF)-β/bone morphogenetic protein (BMP) signaling pathway [11–14] and also involves in regulation of other important biological pathways, such as the non-canonical Wnt pathway and the mitogen-activated protein kinase pathway. Therefore, multiple functions of Smurf1 has been demonstrated in bone homeostasis [11, 12, 14], embryogenesis [15] and autophagy [16]. Smurf1 uses the WW domains to capture Smads, characterized the PY motif, and controls TGF-β/BMP signaling transduction by degrading the components including Smad1, Smad4, Smad5 and Smad7 in the ubiquitination process [11, 14, 17, 18]. Meanwhile, to regulate cell growth and differentiation, Smurf1 also has other substrates in the TGF-β and BMP pathways, including RunX2, RunX3, Tbx6, MEKK2, JunB, and TRAF4 [19–23].

The maintenance of bone homeostasis depends on the dynamic regulation of two processes: bone formation and resorption, in which the osteoblast acts on promoting bone formation and bone increase. The differentiation of mesenchymal stem cells (MSCs) into osteoblasts and osteogenesis relies on the BMP signaling. As the components and related transcription factors of the BMP pathway Smads, MEKK2, RunX2 and JunB are crucial in regulation the differentiation and growth of osteoblasts. Therefore, as the negative factor of BMP pathway, Smurf1 is closely related to the osteoblast differentiation and postnatal bone formation. *Smurf1* ^-/-^ mice display bone mass increase in an age-dependent manner [12, 22].

In consideration of E3 activity and the physiological functions of Smurf1, targeting this negative regulator of BMP pathway will specifically regulate E3 activity, control signal transmission and affect pathological manifestations, which become a candidate strategy in future bone metastasis disease therapies. Up to now, a series of studies have shown that certain activators could enhance the E3 activity of Smurf1and augment Smurf1-mediated ubiquitination, such as casein kinase 2 interacting protein-1 (CKIP-1) [22, 24] and Cdh1, which is identified as the activator of anaphase-promoting complex(APC) [10]. Furthermore, these activators reduce cellular responses to TGF-β/BMP signaling, and depress the bone formation process and attenuate osteogenetic activity. Otherwise, a cullin E3 ligase complex named SCF^FBXL15^ could ubiquitinate Smurf1 and induce its proteasomal degradation [25]. As SCF^FBXL15^ downregulates the protein level of Smurf1, it leads to the decline of E3 activity and increase of osteogenesis.

The critical role of Smurf1 in the bone formation regulation has been extensively investigated and the relevant substratesin the BMP pathway also has been discovered. However, how to hold back Smurf1 receiving Ub and then regulate BMP pathway and osteoblastic activity is little known. In this study, we simulated the Ub binding region of Smurf1 and identified two small molecule compounds via computer virtual screening. They specifically target the HECT domain of Smurf1 and interrupt Ub-Smurf1 contact, thus inhibit the degradation of Smad1/5. Notably, this work further demonstrate these two compounds strengthen the bone synthesis ability by promoting BMP signal transduction. So far as we know, this is the first time to identify small molecule compound which specifically targets the HECT domain of Smurf1 ligase.

## RESULTS

### Computer virtual screen compounds aim at HECT domain of Smurf1

**Figure 1:**
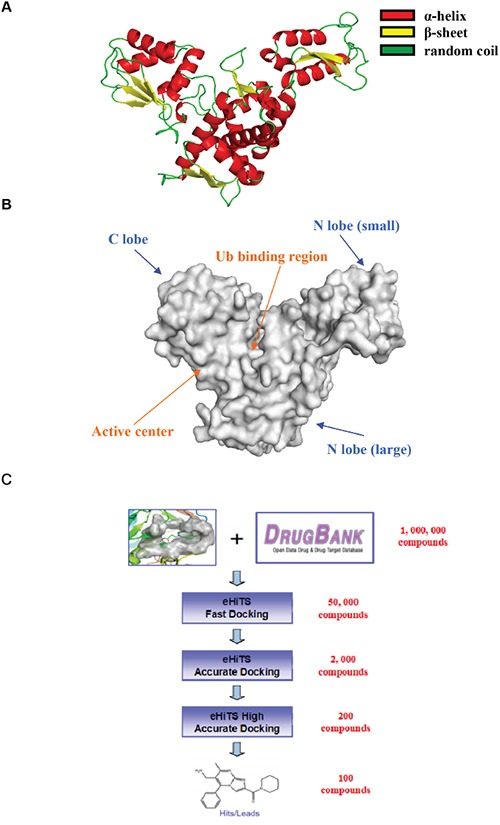
Computer virtual screen compounds targeting the HECT domain of Smurf1 **A**. The protein modeling of Smurf1 HECT domain. The α-helix (red), β-sheet (yellow) and random coil (green) were labeled in HECT domain. **B**. The surface structure of Smurf1 HECT domain. The Ub binding region (orange arrow), active center (orange arrow) and two lobe (blue arrows) were point out by different arrows. **C**. Work-flow of computer virtual screening.

The structure of Smurf1 WW domains (WW1 and WW2) and a phospholipid binding C2 domain have been identified and analyzed, however the three-dimensional structure of Smurf1 catalytic HECT domain remains unclear. Given that both Smurf1 and Smurf2 belong to Nedd4 family, and their amino acid sequence homology of HECT domain is more than 90%, the structure of this domain was obtained by protein modeling performed on PyMOL and referred to the counterpart domain of Smurf2 (Figure 1A). Based on the structure, we defined a concave region on the HECT domain, surrounded by Asn431, Tyr439, Asn481 and Gln653, as a hydrophobic pocket which is likely to key area for its combination with ubiquitin (Figure 1B). By computer high-throughput virtual screening, we acquired about 100 compounds that might target HECT domain of Smurf1 from more than one million available small molecule compounds (Figure 1C).

### Candidate compounds B06 and B75 enhance osteoblast differentiation via activating BMP signaling

**Figure 2:**
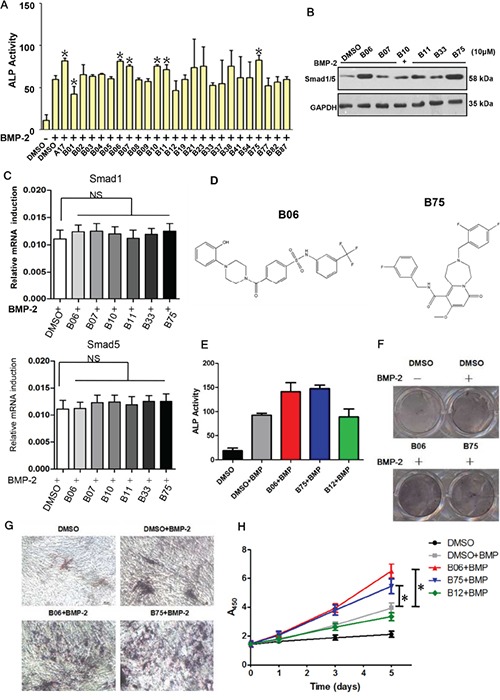
Candidate compounds B06 and B75 promotes the bone synthesis ability via activating BMP signaling **A**. The effection of candidate compounds to ALP activity. C2C12 cells were deal with DMSO (0.1%) or compound (2μM), then it were stimulated by BMP-2 (50ng/ml) after 1h, ALP activity test wound be executed after 48h. **B**. The effection of candidate compounds to the expression of Smad1/5 protein. C2C12 cells were deal with DMSO (0.1%) or compound (10μM), then it were stimulated by BMP-2 (50ng/ml) after 1h, the proteins expression wound be detected after 8h by western blot. All of the above data points were determined in triplicate and showed with the mean±SD (*:p≤0.05, t-test). **C**. C2C12 cells were treated B06 and B75 at 2 mM, while BMP-2 was used at 50 ng/ml. Data points were determined in triplicate and showed with the mean±SD (*:p≤0.05, t-test). **D**. Chemical structures of B06 and B75. **E**. Selective compounds enhance BMP-2 induced ALP activity. C2C12 cells were dealed with DMSO (0.1%) or compound (2μM), then it were stimulated by BMP-2 (the concentration is 100ng/ml) after 1h. ALP activity test was performed after 48h. Data points were determined in triplicate and showed with the mean±SD (*:p≤0.05, t-test). **F**. Selective compounds enhance BMP-2 induced ALP content. C2C12 cells were dealed with DMSO (0.1%) or compound (2μM), then it were stimulated by BMP-2 (100ng/ml) after 1h, ALP colouration test was performed after 48h. **G**. ALP staining results were shown under microscope. **H**. Selective compounds can obvious promote BMP-2 induced cells proliferation. Data points were determined in triplicate and showed with the mean±SD (*:p≤0.05, t-test).

By high-throughput virtual screening and compounds skeleton classification, 24 representative compounds were selected in the top 100 scoring ones for the next preliminary selection (Supplementary Tables S1 and S2). Since Smurf1 could reduce cellular responses to bone morphogenetic protein (BMP) via triggering proteasomal destruction of Smad1 and Smad5, we made alkaline phosphatase (ALP) activity as chief standard in the preliminary selection, since it is one of the most canonical marker that can reflect bone formation ability in bone anabolism. For the subsequent assays, we selected mouse myoblasts cell line C2C12, which possesses characteristic of mesenchymal stem cell and respond to BMP signal. We measured ALP activity after treatment with the compound (2 μM) and BMP-2 (50μM) in the C2C12 cells. A previously identified Smurf1 WW domain-targeting compound named A17 was used as a positive control in these assays [26]. The results showed that B06, B07, B10, B11 and B75 improved ALP activity compared with negative control DMSO (Figure 2A). These candidate compounds were further screened with analysis of Smad1/5 protein levels. After treatment with each of the examined compounds (10 μM) and BMP-2 (50 μM), the compounds B06 and B75 obviously up-regulated the protein level of Smad1/5 (Figure 2B). In addition, the mRNA levels of Smad1 and Smad5 were not significantly changed under the same treatment conditions with these compounds, suggesting that B06 and B75 specifically affect Smad1/5 protein level (Figure 2C). Meanwhile, we found that both B06 and B75 fit the rules of Lipinski and possess distinct properties (such as structure) (Figure 2D and Supplementary Table S3). Based on the above results, we speculated that the small molecule compounds B06 and B75 might be the best candidates among the examined compounds. To verify the effects of B06 and B75 in promoting bone synthesis in C2C12 cells, we performed ALP activity assays again under BMP-2 stimulation with a high-does treatment (50 μM). Compared with the control group, both B06 and B75 enhanced ALP activity, whereas another compound B12 did not (Figure 2E). Furthermore, we performed ALP staining assays, and confirmed that they were able to increase the intracellular ALP content under BMP-2 stimulation (Figure 2F and 2G). In order to investigate the effect of B06 and B75 on C2C12 cell proliferation, we performed WST-1 cell proliferation and cell toxicity test. The results showed that both B06 and B75 can promote C2C12 cells proliferation, and their toxicity was low (Figure 2H). Taken together, these data suggest B06 and B75 can enhance the osteoblast differentiation of C2C12 cells.

### B06 and B75 inhibit Smurf1-mediated Smad1/5 ubiquitination and degradation

**Figure 3:**
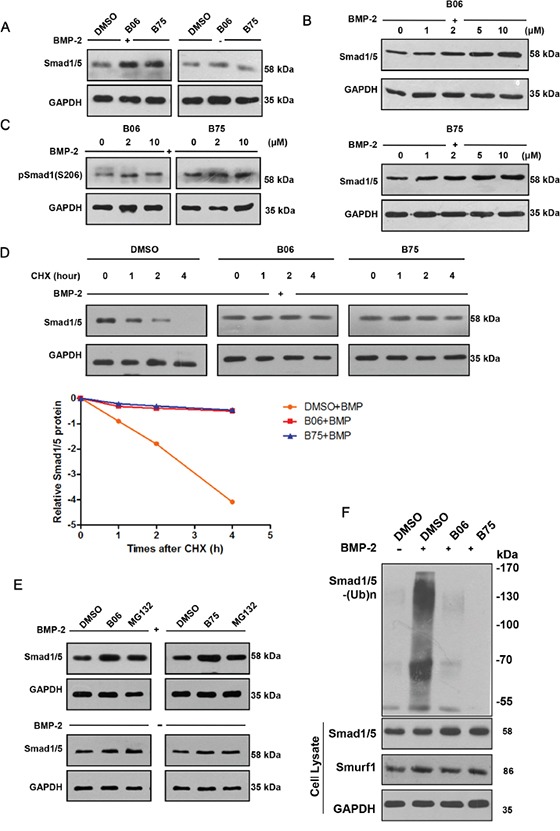
B06 and B75 inhibit Smurf1-mediated Smad1/5 ubiquitination and degradation **A**. C2C12 cells were treated with B06 and B75 (2μM) together with rhBMP-2 (50 ng/ml) or not. GAPDH were used as loading controls. **B**. Selective compounds can increase the Smad1/5 protein level. C2C12 cells were dealed with DMSO (0.1%) or compound (the concentration is 1μM, 2μM, 5μM and 10μM), then it were stimulated by BMP-2 (50 ng/ml) after 1h, the proteins expression was detected after 8h by WB. **C**. Selective compounds can increase Smad1 phosphorylation (S206) level. C2C12 cells were dealed with compound (2 μM), and stimulated by BMP-2 (50 ng/ml); after 1h, the p-Smad1(S206) protein level was detected after 8h by WB. **D**. Selective compounds prolonged the half-time of Smad1/5 protein. C2C12 cells were dealed with DMSO (0.1%) or compound (2 μM), then stimulated by BMP-2 (50 ng/ml) and CHX (10 μg/ml); after 1h, the Smad1/5 protein expression was detected by WB after the indicated times (0, 1, 2 and 4h). The data were analyzed through software Image J and GraphPad Prism. **E**. Detection of Smad1/5 protein level following selective compounds or proteasome inhibitor (MG132) treatments. **F**. Selective compounds impeded the ubiquitination of Smad1/5. C2C12 cells were treated B06 and B75 at 2 μM, while MG132 and rhBMP-2 were used at 20 mM and 50 ng/ml. GAPDH were used as loading controls.

We confirmed that the selective compounds B06 and B75 upregulated the Smad1/5 protein levels (Figure 3A) and both compounds increased Smad1/5 protein level in a dose-dependent manner (Figure 3B). Since S206 of Smad1 was phosphorylated modified in BMP-2 signaling pathway, we tested the expression of p-Smad1 (S206) and confirmed that B06 and B75 are able to upregulate its level (Figure 3C). We also performed protein decay experiment and the subsequent test revealed that B06 and B75 could prolong the half-life of endogenous Smad1/5 under BMP-2 stimulation (Figure 3D). Next, we compared the effect of selective compounds and proteasome inhibitor MG132. Under BMP-2 stimulation, B06 and B75 were able to upregulateSmad1/5 protein level which effect was similar to that of MG132 (Figure 3E). Furthermore, we performed a Smad1/5 ubiquitination assay *in vivo* to test whether B06 and B75 stabilize Smad1/5 protein level via inhibiting Smurf1-mediated Smad1/5 ubiquitination. The result showed that B06 and B75 strongly inhibited Smad1/5 ubiquitination under rhBMP-2 stimulation compared with control (Figure 3F).

### B06 and B75 interrupt interaction between Smurf1 and Ub but not Smurf1 and Smad1/5

**Figure 4:**
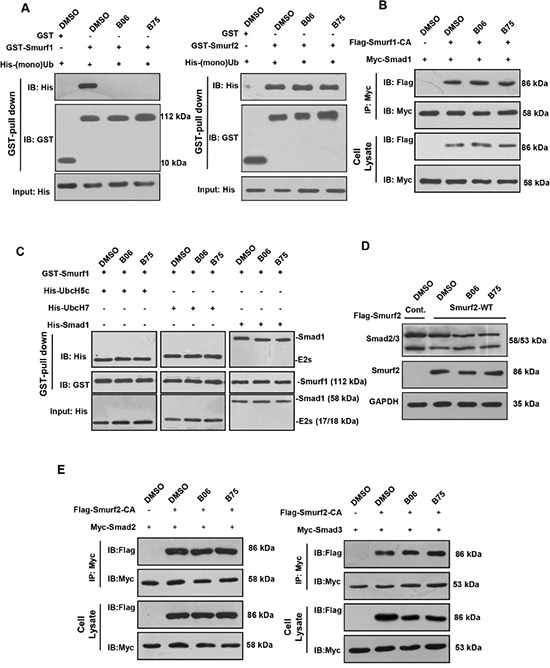
B06 and B75 compounds interrupt the interaction between Smurf1 and Ub **A**. GST pull-down assays were performed to show that GST-tagged Smurf1 and Smurf2 directly interacts with mono-Ub *in vitro*, and B06 and B75 specifically inhibit Smurf1-Ub binding. **B**. Selective compounds had no significant effects on Smurf1-Smad1 interaction. 293T cells were co-transfected with Flag-empty vector and Myc-Smad1 (lane 1) or Flag-Smurf1-CA and Myc-Smad1 (lanes 2–4) plasmids. For inhibitors administration, cells were treated with B06 and B75 at 2μM. **C**. Selected compounds do not interrupt Smuf1-E2 contact. Prokaryotic expressed proteins were purified and employed in GST-pull down. B06 and B75 were used at 10mM. Note that cropped blots are shown here. **D**. 293T cells were transfected Flag-empty vector (lane 1) and Flag-Smurf2 (lane 2). For inhibitors administration, cells were treated with B06 and B75 at 2μM (lanes 3-4). **E**. Left: 293T cells were co-transfected with Flag-empty vector and Myc-Smad2 (lane 1) or Flag-Smurf2-CA and Myc-Smad2 (lanes 2-4) plasmids. Right: 293T cells were co-transfected with Flag-empty vector and Myc-Smad3 (lane 1) or Flag-Smurf2-CA and Myc-Smad3 (lanes 2-4) plasmids. For inhibitors administration, cells were treated B06 and B75 at 2μM. Note that cropped blots are shown here.

Given the screen rationale, we next investigated whether B06 and B75 weaken or block the direct interaction between Smurf1 and Ub, the binding assay *in vitro* was performed. The pull-down results showed that single Ub protein can be readily copurified with GST-Smurf1, and incubation with B06 and B75 interrupted Smurf1 and Ub binding. Since the amino acid sequence homology of Smurf1 and Smurf2 HECT domains are more than 90% and Smurf2 also contains a Ub-binding region to capture Ub molecules, we tested the effect of B06 and B75 on interaction between Smurf2 and Ub. However, B06 and B75 did not interrupt the interaction between Smurf2 and Ub (Figure 4A). We further examined the possible impacts of the two compounds on Smurf1-Smad and Smurf1-E2 interaction. The Smurf1-Smad1 interaction assay was performed that exogenous Smad1 was transfected into HEK293T cells with Smurf1-CA mutant, which abolishes ubiquitin ligase activity and fails in ubiquitination by changing the HECT domain crucial site Cys699 to an Ala. However, this point mutant still reserves binding ability to its interacting proteins. Co-immunoprecipitation of Smad1 showed that both selective compounds B06 and B75 had no effect on Smurf1 interaction with Smad1 (Figure 4B). Similarly, an *in vitro* binding assay was performed between Smurf1 and its E2s, UbcH5c and UbcH7, which interact with the HECT domain of Smurf1 and deliver the ubiquitins onto it. The result showed that selected compounds did not interrupt Smuf1-E2 interaction (Figure 4C). In conclusion, B06 and B75 specifically interfere with the interaction between Smurf1 and Ub but not Smurf1 and Smad1/5. We also tested the possible effect of the compounds on Smurf2 with the substrates Smad2/3. The results showed that Smurf2 downregulated the protein level of Smad2/3, as expected, however, B06 and B75 had no inhibitory effects on the degradation (Figure 4D). Subsequently, the effect of both compounds on the interactions of Smurf2-Smad2/3 were tested via *in vivo* co-immunoprecipitation assays. The results showed that B06 and B75 could not interrupt Smurf2-Smad2 or Smurf2-Smad3 interactions (Figure 4E), indicating that both compounds might act specifically on Smurf1.

### B06 and B75 control Smad1/5 and other targets in a Smurf1-dependent manner

**Figure 5:**
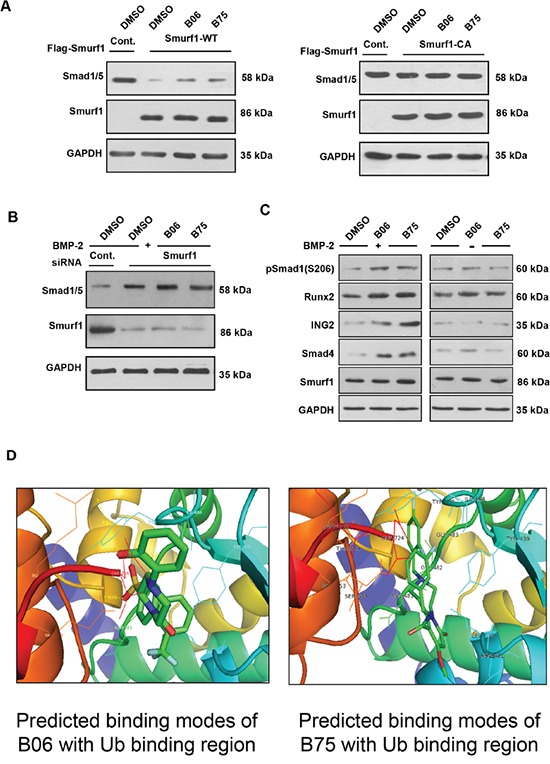
B06 and B75 control Smad1/5 and other targets in a Smurf1-dependent manner **A**. 293T cells were transfected with the indicated plasmids. For inhibitors administration, cells were treated with B06 and B75 at 2μM. **B**. Smurf1 was knocked down in C2C12 cells (lane 1: control siRNA, lanes 2–4: mouse Smurf1 siRNA). For inhibitors administration, cells were treated with B06 and B75 at 2μM, while rhBMP-2 was used at 50 ng/ml. GAPDH were used as loading controls. Note that cropped blots are shown here. **C**. Effects of selective compounds on other substrates of Smurf1. **D**. Predicted binding mode of B06 with the defined Ub binding region. Key residues in the region were labeled in different colours. The distances (angstrom) of hydrogen bonds donors and receptors were noted in numerical values and yellow dotted lines. Predicted binding modes of B75 with the defined Ub binding region. Key residues in the region were labeled in black.

*In vivo* assays revealed that B06 and B75 could elevate Smad1/5 when cells were pre-transfected wild type Smurf1 but not the C699A (Smurf1 CA) mutant (Figure 5A). To identify whether the compounds affect Smad1/5 in a Smurf1-dependent manner, we knocked down Smurf1 by specific siRNA under rhBMP-2 stimulation. We found that neither B06 or B75 could elevate Smad1/5 protein level (Figure 5B), indicating the dependence of Smurf1. The fact that B06 and B75 interrupt the interaction between Smurf1 and Ub implies that they might inhibit Smurf1-mediateddegradation of other substrates. To verify this point, we examined other reported substrates of Smurf1 besides Smad1/5. It turned out that both B06 and B75 upregulated the protein level of Runx2, Smad2/3, Smad4 and ING2 (Figure 5C), although their binding modes with Smurf1 are incompletely identical. That confirmed that B06 and B75 specifically inhibit Smurf1-mediated ubiquitin-proteasomal degradation rather than impairs the substrate binding. Finally, we predicted the binding modes of B06 and B75 with the defined Ub binding region for intensive research (Figure 5D).

## DISCUSSION

In this study, we screened small molecules which specifically targeted the ubiquitin-binding site within Smurf1 HECT domain. A typical HECT domain consists of two lobes: N-lobe interacts with the E2 and C-lobe contains the active-site cysteine that forms the thioester bond with ubiquitin [27]. Interestingly, Smurf1 and Smurf2 also contain a non-covalent ubiquitin binding region within the N-lobe, which is required for their E3 ligase activity. We established the strategy that screened compounds through computer virtual and found the best match compounds B06 and B75 inhibiting the combination between Smurf1 and Ub could enhance BMP signaling and promote C2C12 cells differentiation and proliferation. This strategy specifically targeted the Ub binding region of HECT domain, and did not affect the interaction of Smurf1 and E2. Therefore, it is a novel strategy to inhibit the ligase activity and control the Smurf1-mediated degradation.

As mentioned above, Smurf1 controls the turnover of components of BMP signaling cascades, which is a negative regulator of osteoblast differentiation and bone formation. The identified mechanisms regulating Smurf1 include the transcriptional and post-transcriptional level. At the transcriptional level, tumor necrosis factor (TNF) increases Smurf1 expression by elevating Smurf1 transcription and inhibits osteoblastic bone formation [28–30]. At post-transcriptional level, nuclear protein kinase CK1α stabilizes the expression of Smurf1 by regulating the expression of vertebrate-specific pre-mRNA binding protein heterogeneous nuclear ribonucleoprotein (hnRNP-C) [31]. In mesenchymal stem cells, microRNA-17 directly targeting Smurf1 3’UTR hinder the E3 ligase expression and enhance osteogenesis [30]. Moreover, we have previously shown that Smurf1 expression and activity are also regulated by CKIP-1, an activator augments the E3 ligase activity by targeting its linker region between the two WW domains, thereby promoting Smurf1-mediated ubiquitination [24]. We subsequently established a new approach targeting CKIP-1 to treat metabolic skeletal disorders, which uses (AspSerSer)6-liposome to deliver siRNAs specifically to bone-formation surfaces. This target delivery system selectively stimulates bone formation without affecting bone resorption [32]. Besides CKIP-1, others find that Cdh1, another activator interacting with the C2 and WW1 domains of Smurf1, disrupts the auto-inhibitory Smurf1 dimers and promotes the E3 ligase activity of Smurf1 (10). On the other hand, a series of E3 ligases including SCF^FBXL15^, and Smurf2 were shown to trigger ubiquitin–proteasomal degradation of Smurf1 [25, 33]. Recently, we analyzed the reported co-structure of Smad1 and Smurf1 WW domains interaction, which affects Smurf1 activating Smad1, and identified a binding hydrophobic pocket providing the position for this interaction. Ultimately, we discovered the small molecule compounds A01 and A17 blocking the WW1 domain interacting with Smad1 based virtual screening [26]. We draw inspiration from this successful strategy and design the similar and distinctive scheme for screening the small molecule compounds targeting Ub binding region of Smurf1 instead of the interaction between Smurf1 and substrates.

A previous study showed that both Smurf1 and Smurf2 HECT domains exihibit ubiquitin-binding surfaces for promoting substrate poly-ubiquitination [34]. Mutations in Tyr459 and Gly404 weaken the E3 activity of Smurf2, which indicates these two sites in the hydrophobic surface of the N lobe might exist Ub-binding region to capture Ub molecules for Smurf2-mediated ubiquitination as well as Smurf1. So far nine E3 ligase have been identified belonging to the Nedd4 family: Nedd4, Nedd4L, WWP1, WWP2, NEDL1, NEDL2, Itch, Smurf1 and Smurf2. On one hand, they are jointly involved in regulation of a distensible substrate network. On the other hand the multiple functions of these ubiquitin ligase have been explored in different biological and physiological process include embryonic development, the immune response and tumor invasion. Nedd4 as the specific E3 ligases directs ubiquitination of the tumor suppressor PTEN and positively regulates cell growth and development [8, 35]. Nedd4L, reveals similar functions with Nedd4, such as in viral budding and endocytosis [36, 37]. In addition, Nedd4L also targets Smad2, Smad4 and the TGF-β receptor for ubiquitination of these substrates [38]. Itch plays a vital role in Th2 cell differentiation [34]. WWP1 promotes ubiquitination of RunX2 and have been identified a negative regulators of bone formation [39]. Furthermore, other Nedd4 family members have the partial overlapping functions in regulating TGF-β and BMP pathways besides Smurf1. Therefore, whether these members possess the Ub binding region or not? If they had, we would apply the novel strategy we established for searching the small molecule compounds which could disrupt the interaction between them and Ub, and control the relevant biological processes.

The finding of the function of B06 and B75 on inhibiting Smurf1-Ub contact also raises certain questions which should be addressed in future studies. For example, we still need develop an experimental animal model to test and verify the effects of the compounds on bone mass *in vivo*. In addition, whether the compounds are toxic or have undesirable side effects on organism is also worthy of further investigation.

In conclusion, our findings reveal a novel mechanism and strategy to search the small molecule compounds which target Ub binding region of the HECT E3 ligase Smurf1 and block the interaction between HECT domain and Ub. The study provides a new and original strategy to control the activity of HECT E3 ligase.

## MATERIALS AND METHODS

### Cell culture and reagents

mouse myoblast cell line C2C12 cells and human embryonic kidney cells 293T cells were cultured in DMEM supplemented with 5% fetal bovine serum (FBS). Cells were transfected with TurboFect (R0531, Thermo Scientific). BMP-2 was purchased from PeproTech. Compounds were purchased from JK chemical. Proteasome inhibitor MG132 was obtained from Sigma. FBS was obtained from Hyclone. DMEM was purchased from Corning.

### Antibodies

All antibodies were purchased as follows: Antibodies against Smurf1 (ab38866, Abcam), anti-Smad1/5 (ab75273, Abcam), anti-Smad2/3 (#8685, CST), anti-Smad4 (#9515, CST), anti-pSmad1(Ser206) (#13820, CST), anti-Runx2 (ab23981, Abcam), anti-ING2 (ab109504, Abcam), anti-Myc(MBL), anti-GST(MBL), anti-His (MBL), anti-HA (MBL), anti-GAPDH (MBL), anti-Flag (MBL) and mouse/rabbit IgG (Santa Cruz).

### Computer virtual screening

The structures of Smurf1 HECT domain was obtained by protein modeling performed on PyMOL, referred to the counterpart domain of Smurf2. Afterwards, a large compounds pool was be comprised of form four chemistry industrial companies: InterBioScreen Ltd., ChemBridge Corporation, ENAMINE Ltd. and Life Chemicals Inc. Then, all compounds were evaluated the drug ability by ADMET Predictor (Simulations Plus Inc. USA), on which a cutoff line was set to eliminate the compounds with ADMET risk score of ≤2, to shrink this pool to smaller virtual library of about one million compounds. The target pocket was defined by the docking program eHiTS (SimBioSys Inc. Canada), and the virtual ligand auto-docking was performed by three hierarchical steps: (1). Fast docking. The step abated the pool to 50,000 compounds. (2). Accurate docking. The step abated the pool to 2,000 compounds. (3). High accuracy docking. The step abated the pool to 200 compounds. The eHiTS score (log Kd) of each ligand was generated simultaneously after these procedures. The docking conformation of each ligand and the pocket was visualized by CheVi (SimBioSysInc Canada). Finally, we obtained the top 100 scoring ones.

### Western blot

Whole cell lysates were prepared according to the manufacturer's instructions. Protein samples were subjected to SDS-PAGE, and electrophoresed proteins were subsequently transferred onto a PVDF membrane. Membranes were blocked with 5% nonfat dry milk in Tris-buffered saline with 0.1% Tween20 and incubated with the indicated primary antibody, which was followed by incubation with an HRP-conjugated secondary antibody. Immune complexes were visualized with the Supex reagent, and luminescence was detected. Before assays, cells were cultured with the administration of compounds and BMP-2. In addition, cells were harvested 8 hours after compounds administration.

### Co-immunoprecipitation assay

Transfection was performed using TurboFect(R0531, Thermo Scientific) following the manufacturer's instructions. After 48h harvested, cells were lysed in HEPES lysis buffer (20 mM HEPES, pH 7.2, 50 MmNaCl, 0.5% Triton X-100, 1 mMNaF and 1 mM DTT) supplemented with protease inhibitor cocktail (Roche). Immunoprecipitations were performed using the indicated primary antibody for 3-4h and protein A/G-agarose beads (Santa Cruz) overnight at 4°C. The resulting immunoprecipitates were washed at least three times in HEPES lysis buffer. Lysates and immunoprecipitates were examined using the indicated primary antibodies followed by detection with the related secondary antibody and the SuperSignalchemiluminescence kit (Thermo).

### Protein half-life assay

For Smad1/5 half-life assay, when C2C12 cells in 20mm plates reached about 60% confluence. Cells were cultured with the administration of compounds and BMP-2. Eight hours later, cells were treated with the protein synthesis inhibitor cycloheximide (10 μg/ml) for the indicated durations before harvest.

### *In vivo* ubiquitination assay

C2C12 cells were cultured in 100mm medium reached about 60% confluence with the administration of compounds and BMP-2. After 12 hr, cells were treated with 20 μM proteasome inhibitor MG132 (Calbiochem) for 8 hr. The cells were washed with PBS, pelleted, and lysed in 0.4 ml of HEPES buffer (20 mM HEPES, pH 7.2, 50 mMNaCl, 1 mMNaF, 0.5% Titon-X100) plus 0.1% SDS, 20μM MG132 and protease-inhibitor cocktail. The lysates were centrifuged to obtain cytosolic proteins. Briefly, individual samples were incubated with anti-Smad1/5 antibody (Abcam) for 3 hand protein A/G-agarose beads (Santa Cruz) for a further 8 h at 4°C. Then the beads were washed thrice with HEPES buffer. The proteins were released from the beads by boiling in 40 ml of 2× SDS-PAGE sample buffer for 10 min. Ten microliters of the samples were subjected to immunoblot against anti-HA monoclonal antibody (MBL) in individual experiments.

### GST pull-down

To detect the direct binding of Smurf1 with Ub, bacteria-expressed GST, GST-Smurf1 proteins were immobilized on Glutathione-Sepharose 4B beads (Amersham Biosciences) and washed, and then beads were incubated with His-Ub for 8 h at 4°C under rotation. Beads were washed with GST-binding buffer (100 mMNaCl, 50 mMNaF, 2 mM EDTA, 1% NP-40 and protease inhibitor mixture) and proteins were eluted, followed by immunoblotting. The direct binding assay of Smurf1-Smad1, Smurf1-UbcH5c, Smurf1-UbcH7, Smurf2-Ub are the same with this assay.

### ALP activity assay and ALP staining

Cells were cultured in 100mm medium for three days and administrated with compounds and rhBMP-2 on the day 3. ALP activity was examined by Alkaline Phosphatase, Diethanolamine Detection Kit (Sigma-Aldrich) following the manufacturer's protocols. ALP staining was performed by BCIP/NBT Alkaline Phosphatase Color Development Kit (Beyotime) following the manufacturer's protocols. Cells were stained on the 6th day.

### Cell counting assay

Cell counting assay was performed by WST-1 Cell Proliferation and Cytotoxicity Assay Kit (Beyotime) following the manufacturer's protocols.

### Molecular visualization and statistical analysis

Protein and compounds structures results were visualized and plotted by PyMOL (DeLano) and statistical analysis was performed with student's t-test by SPSS statistics 17.0.

## SUPPLEMENTARY MATERIALS TABLES




