# Supplementary material for: Selective compounds enhance osteoblastic activity by targeting HECT domain of ubiquitin ligase Smurf1
Source: Oncotarget. 2016 Jul 18;8(31):50521–33. doi: 10.18632/oncotarget.10648 (PMC5584161; doi:10.18632/oncotarget.10648)
Supplement: Supplementary file 1 [file oncotarget-08-50521-s001.pdf]

Selective compounds enhance osteoblastic activity by targeting HECT domain of ubiquitin ligase Smurf1

SUPPLEMENTARY TABLES

Supplementary Table S1: The skeleton category and molecular weight of candidate compounds

Skeleton Category	Typical Compounds	Molecular Weight	Skeleton Category	Typical Compounds	Molecular Weight	Skeleton Category	Typical Compounds	Molecular Weight
1	B10<br>B54<br>B77	466,<br>464,<br>444	8	B33<br>B37	465,<br>413	17	B75	471
2	B08	556	9	B09	439	19	B04	477
3	B01<br>B23	428,<br>479	10	B21	490	21	B05	489
4	B03	474	12	B87	497	23	B41	481
5	B06	505	13	B02	538	others	B07<br>B11<br>B12<br>B38	457,<br>438,<br>465,<br>475
6	B19	512	15	B82	486			

Supplementary Table S2: Chemical structure and eHiTS score of candidate compounds

See Supplementary File 1

Supplementary Table S3: Basic information of selective compound B06 and B75

See Supplementary File 1
