# Supplementary material for: Selective compounds enhance osteoblastic activity by targeting HECT domain of ubiquitin ligase Smurf1
Source: Oncotarget. 2016 Jul 18;8(31):50521–33. doi: 10.18632/oncotarget.10648 (PMC5584161; doi:10.18632/oncotarget.10648)

**Table S2 Chemical structure and eHiTS score of candidate compounds**


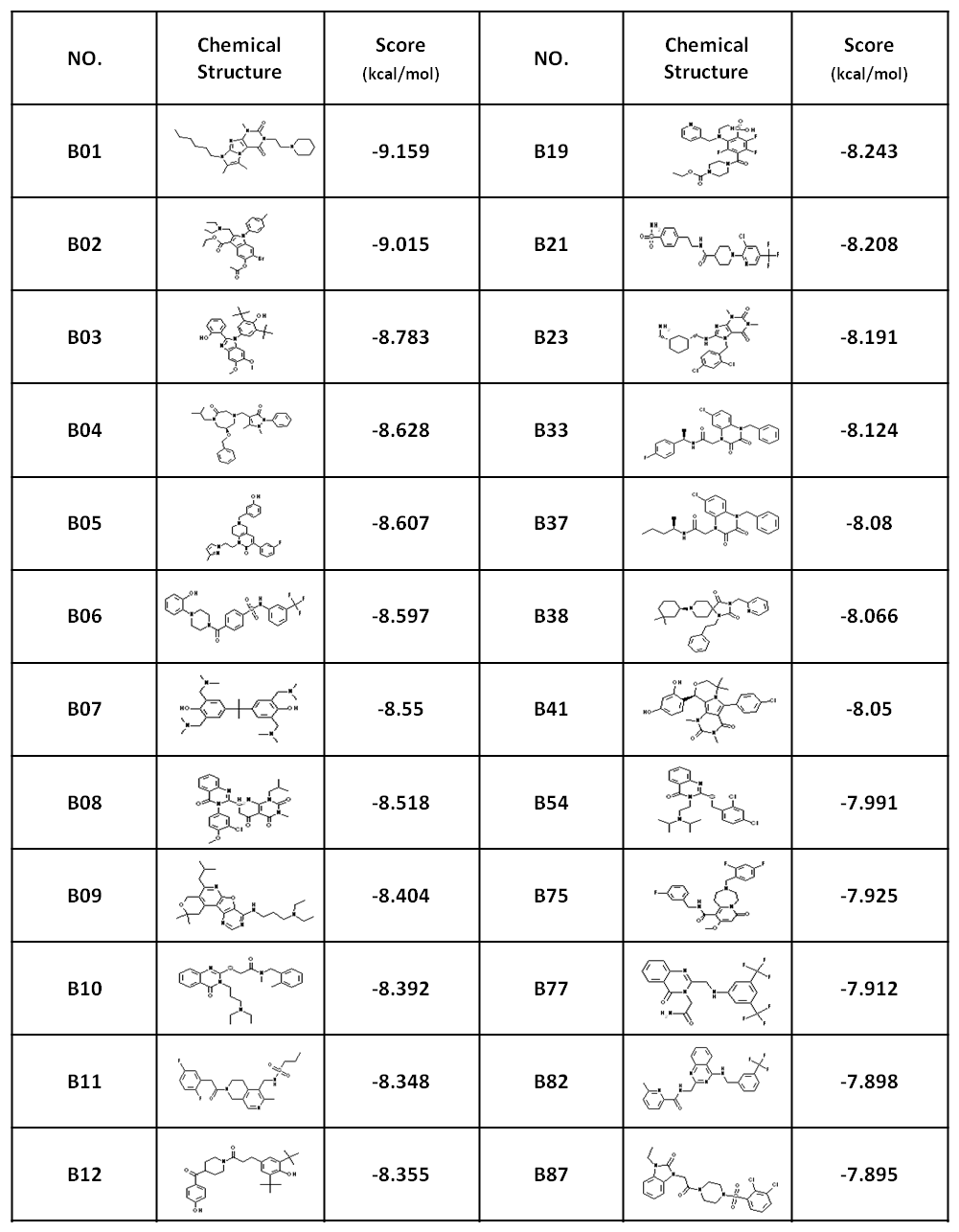


**Table S3 Basic information of selective compound B06 and B75**


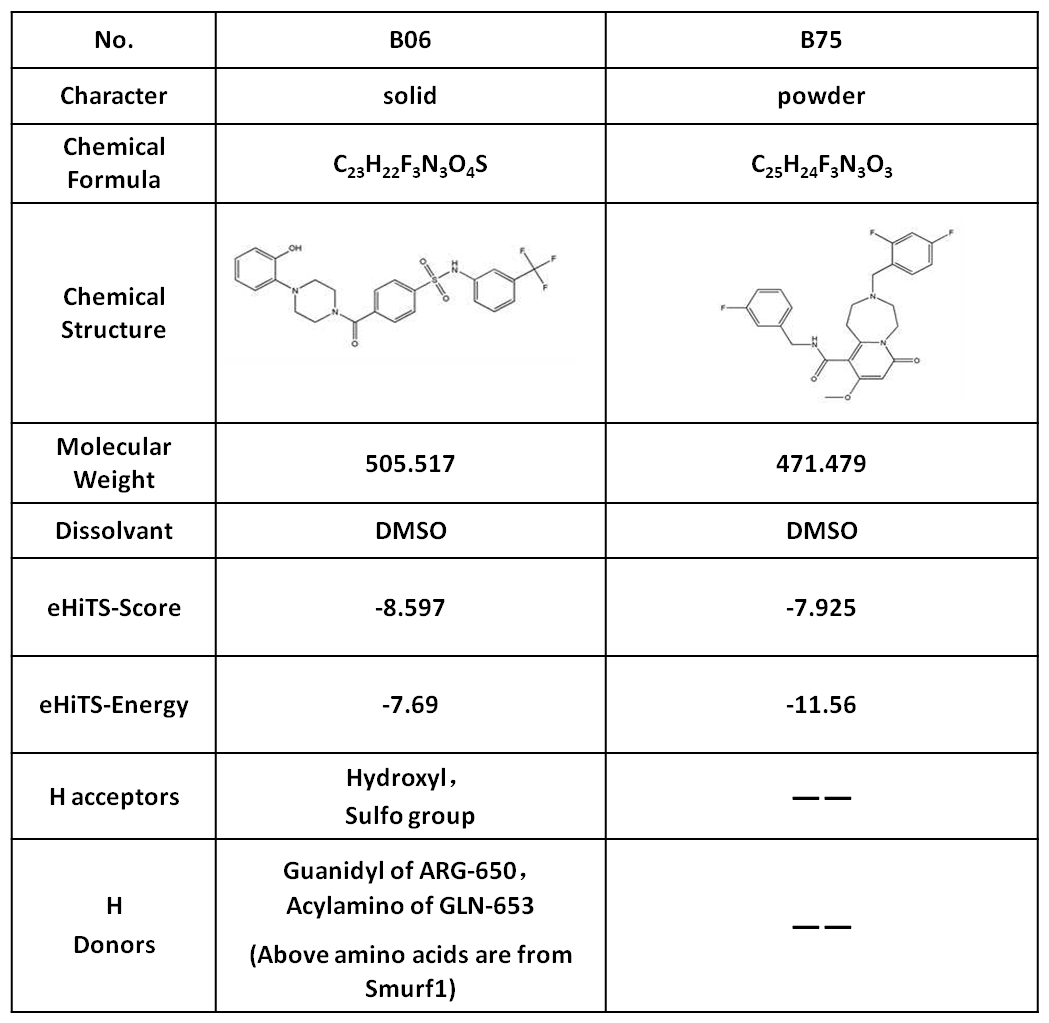

Supplement: Supplementary file 2 [file oncotarget-08-50521-s002.docx]
